# EuroFlow-Based Flowcytometric Diagnostic Screening and Classification of Primary Immunodeficiencies of the Lymphoid System

**DOI:** 10.3389/fimmu.2019.01271

**Published:** 2019-06-13

**Authors:** Jacques J. M. van Dongen, Mirjam van der Burg, Tomas Kalina, Martin Perez-Andres, Ester Mejstrikova, Marcela Vlkova, Eduardo Lopez-Granados, Marjolein Wentink, Anne-Kathrin Kienzler, Jan Philippé, Ana E. Sousa, Menno C. van Zelm, Elena Blanco, Alberto Orfao

**Affiliations:** ^1^Department of Immunohematology and Blood Transfusion, Leiden University Medical Center, Leiden, Netherlands; ^2^Department of Immunology, Erasmus MC, Rotterdam, Netherlands; ^3^Department of Pediatrics, Leiden University Medical Center, Leiden, Netherlands; ^4^Department of Pediatric Hematology and Oncology, University Hospital Motol, Charles University, Prague, Czechia; ^5^Department of Medicine, Cancer Research Centre (IBMCC, USAL-CSIC), Cytometry Service (NUCLEUS), University of Salamanca (USAL), Institute of Biomedical Research of Salamanca (IBSAL), Salamanca, Spain; ^6^Biomedical Research Networking Centre Consortium of Oncology (CIBERONC), CB/16/12/00233, Instituto Carlos III, Madrid, Spain; ^7^Institute of Clinical Immunology and Allergology, St. Anne's University Hospital Brno, Masaryk University, Brno, Czechia; ^8^Department of Immunology, Hospital Universitario La Paz, Madrid, Spain; ^9^Experimental Medicine Division, Nuffield Department of Medicine, University of Oxford, Oxford, United Kingdom; ^10^Department of Laboratory Medicine, University Hospital Ghent, Ghent, Belgium; ^11^Faculdade de Medicina, Instituto de Medicina Molecular, Universidade de Lisboa, Lisbon, Portugal; ^12^Department of Immunology and Pathology, Central Clinical School, Alfred Hospital, Monash University, Melbourne, VIC, Australia

**Keywords:** immunodeficiency, immunophenotyping, flow cytometry, diagnosis, classification, EuroFlow, standardization

## Abstract

Guidelines for screening for primary immunodeficiencies (PID) are well-defined and several consensus diagnostic strategies have been proposed. These consensus proposals have only partially been implemented due to lack of standardization in laboratory procedures, particularly in flow cytometry. The main objectives of the EuroFlow Consortium were to innovate and thoroughly standardize the flowcytometric techniques and strategies for reliable and reproducible diagnosis and classification of PID of the lymphoid system. The proposed EuroFlow antibody panels comprise one orientation tube and seven classification tubes and corresponding databases of normal and PID samples. The 8-color 12-antibody PID Orientation tube (PIDOT) aims at identification and enumeration of the main lymphocyte and leukocyte subsets; this includes naïve pre-germinal center (GC) and antigen-experienced post-GC memory B-cells and plasmablasts. The seven additional 8(-12)-color tubes can be used according to the EuroFlow PID algorithm in parallel or subsequently to the PIDOT for more detailed analysis of B-cell and T-cell subsets to further classify PID of the lymphoid system. The Pre-GC, Post-GC, and immunoglobulin heavy chain (IgH)-isotype B-cell tubes aim at identification and enumeration of B-cell subsets for evaluation of B-cell maturation blocks and specific defects in IgH-subclass production. The severe combined immunodeficiency (SCID) tube and T-cell memory/effector subset tube aim at identification and enumeration of T-cell subsets for assessment of T-cell defects, such as SCID. In case of suspicion of antibody deficiency, PIDOT is preferably directly combined with the IgH isotype tube(s) and in case of SCID suspicion (e.g., in newborn screening programs) the PIDOT is preferably directly combined with the SCID T-cell tube. The proposed ≥8-color antibody panels and corresponding reference databases combined with the EuroFlow PID algorithm are designed to provide fast, sensitive and cost-effective flowcytometric diagnosis of PID of the lymphoid system, easily applicable in multicenter diagnostic settings world-wide.

## Introduction

Primary immunodeficiencies (PID) are inherited disorders of the immune system, generally presenting with recurrent, sometimes life-threatening infections. To date, more than 350 genes have been identified that can be mutated in PID patients (1–3). Depending on the genetic defect, one part of the immune system or one cell type can be absent, decreased or dysfunctional. The majority of PID patients (60–65%) have a defect in the lymphoid system, involving B- and/or T-cells alone or in combination with other cells (1–5). Flowcytometric immunophenotyping plays a central role in the diagnostic workup of patients suspected of PID, particularly those involving lymphoid cells (1, 6). An accurate immunophenotypic diagnosis is essential for guiding further functional testing as well as for genetic testing, whether Sanger sequencing or next generation sequencing (NGS) targeted to specific genes, whole exome sequencing (WES), whole genome sequencing (WGS) or combinations thereof (2, 5, 7–10). Considering the clinical heterogeneity in genetically homogeneous disease entities, immunophenotyping has an additional role in understanding the clinical heterogeneity in disease presentation and outcome (11–13). Immunophenotyping can also support treatment decisions and monitoring, such as in case of immunoglobulin (Ig) replacement therapy, hematopoietic stem cell transplantation, and gene therapy (7, 8, 14–18).

In complex diseases with high numbers of affected genes and still many genes to be discovered, some investigators recommend the “Genetics First” approach via targeted NGS, WES, and/or WGS, already at an early phase in the diagnostic process (19, 20). Indeed, in absence of other in-depth diagnostic methods, the “Genetics First” approach has clearly contributed to better classification and more insight in some well-defined disease categories with high genetic diagnosis yields, such as in intellectual disability syndrome, hereditary spastic paraplegias, and neuromuscular disorders (21–24). However, in the complex field of PID most targeted NGS and/or WES studies have genetic diagnosis yields varying from 15 to 30% (25–29), sometimes increasing to 40% or higher, depending on the number of targeted genes (varies from 170 to 571), young age (higher yield in children), high frequency of X-linked diseases, high frequency of families with PID history, and highly consanguineous populations with high frequencies of autosomal recessive diseases, such as in the Middle East and North Africa region (9, 30–34). Importantly, virtually all above-mentioned NGS and/or WES studies did not apply the “Genetics First” approach, because the included PID patients were defined according to the guidelines of the European Society for Immunodeficiencies (ESID) and the International Union of Immunological Societies (IUIS), which include flowcytometric immunophenotyping (2, 5, 6). In fact, the genetic diagnosis yield in immunophenotypically defined PID (sub)categories ranges from >95% in severe combined immunodeficiency (SCID), 85–90% in well-defined agammaglobulinemia patients, ~75% in Hyper IgM syndrome, down to 10–20% among the most frequently occurring PID, such as common variable immunodeficiency (CVID) and immunoglobulin (Ig) isotype deficiencies (5, 35–38).

Clearly, adequate clinical and immunophenotypic characterization of PID patients should guide the diagnostic process; this is supported by the diagnosis and classification guidelines of ESID, IUIS, and Clinical Immunology Society (CIS) (2, 6, 39). Compared to other organ systems, many different genes are involved in the immune system, particularly in mature B-cells during and after germinal center (GC) responses. Use of WES and WGS will detect many allelic immune gene variants, which might not be causally related to disease, implying that significant efforts in immunobiological validation studies will be needed. Furthermore, in-depth immunological and functional studies are essential to define the consequences of genetic defects for the immune system. At least part of these studies will be based on flow cytometry. Especially in case of hypomorphic defects, flow cytometry can help to better understand the effects of the genetic defect on the composition of the lymphoid compartment (11–13, 40, 41). Finally, flow cytometry is an important tool for monitoring of targeted therapies, including cellular therapies (15, 16, 18, 42).

Many PID centers have developed their own local multi-color flowcytometric protocols and antibody panels for diagnosis and classification of PID (8, 14, 43–45). These single-center initiatives have led to a great variability in sample processing, antibody panels, immunostaining procedures, instrument setup, sample measurement and data analysis. In addition, the low incidence and the clinical-immunological heterogeneity of PID hamper prospective multicenter diagnostic validation in large patient series and age-matched healthy controls (43–45). As a consequence, the typical but rare immunophenotypic PID patterns are difficult to compare between centers at the national and international level.

Whilst several recent international efforts have tried to harmonize flowcytometric diagnostics of PID (43–48), they have only been partially successful, mainly because these efforts have been restricted to parts of the full pathway of pre-analytical, analytical and post-analytical procedures, frequently focusing on the antibody panels only. Most proposed antibody panels aim at identification of severe defects in B and T lymphocytes, NK-cells, and/or diagnostic screening for a specific inherited disorder or a specific subgroup of disorders. Examples of such disease-oriented antibody panels are meant for: (i) diagnostic screening and classification of SCID based on quantification of B-, T- and NK-cells with CD3, CD19, and CD56 or CD16; (ii) diagnostic screening of congenital agammaglobulinemia, merely based on enumeration of blood B lymphocytes; (iii) classification of common variable immunodeficiency (CVID) according to the proportion of transitional, non-switched/marginal zone-like (smIgMD^+^) and class-switched (smIgMD^−^), and CD21^dim^ B lymphocytes; (iv) diagnostic screening of DiGeorge patients based on relative blood counts of recent thymic emigrant (RTE) CD4^+^ T-cells, and; (v) quantification of CD4/CD8-double negative TCRαβ^+^ T-cells (DNT) for screening of autoimmune lymphoproliferative syndrome (ALPS) (49, 50). Consequently, such antibody panels do not provide a complete overview of the many distinct subsets of circulating leukocytes, as required for fast, efficient, and cost-effective PID diagnostics.

Several initiatives in other fields of clinical immunology have also lead to consensus antibody panels for harmonized flowcytometric immune monitoring, such as the CLIP study (51), the NIH study (52), the ONE study (53), the Pasteur initiative (54), and the NATURIMMUN consortium (55). These antibody panels allow identification of several subpopulations of B-, T-, and NK-cells (e.g., naïve vs. memory, activated cells, TCRγδ vs. TCRαβ) together with the identification of monocytes, dendritic cells, and granulocytes. However, the proposed antibody combinations do not provide the fully integrated information as needed for diagnosis and classification of PID.

**Figure 1:**
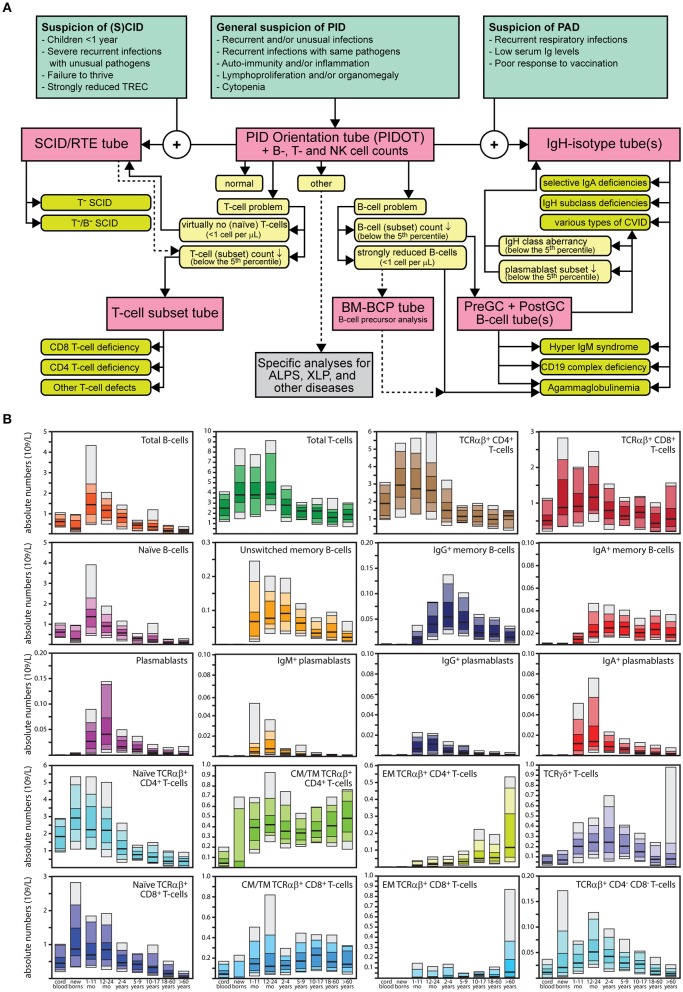
Strategy for flowcytometric immunophenotyping for screening and classification of lymphoid PID. **(A)** EuroFlow algorithm. On the basis of several entries of clinical and laboratory parameters, blood samples of patients suspected to have PID are screened with the 8-color (or 10-color) PID Orientation tube (PIDOT). Based on the obtained results, additional 8-color or 10-color T- and/or B-cell classification tubes are applied in a stepwise fashion, including the BM B-cell precursor (BM-BCP) tube. In case of suspicion of PAD, both the PIDOT and the IgH-isotype tube(s) should be applied together. In case of suspicion of (S)CID and cases with strongly reduced TRECs, both the PIDOT and the SCID/RTE tube can be applied together. See text for detailed description of the stepwise application of the EuroFlow PID tubes. GC, germinal center; PAD, predominantly antibody deficiency; RTE, recent thymic emigrant; SCID, severe combined immunodeficiency. **(B)** Age-related reference values. Absolute counts of all lymphocyte subsets are provided in the format of age-related percentile bars (median; 25–75 percentiles; 10–90 percentiles; 5–95 percentiles). The age groups are: cord blood (*n* = 15), newborns (*n* = 16), 1–11 months (*n* = 19), 12–23 months (*n* = 30), 2–4 years (*n* = 35), 5–9 years (*n* = 28), 10–17 years (*n* = 33), 18–60 years (*n* = 79), and >60 years (*n* = 66). In case of naïve TCRαβ^+^CD4^+^ T-cells, CM/TM TCRαβ^+^CD4^+^ T-cells, EM TCRαβ^+^ CD4^+^ T-cells, TCRγδ^+^ T-cells, naïve TCRαβ^+^CD8^+^, CM/TM TCRαβ^+^CD8^+^ T-cells, EM TCRαβ^+^CD8^+^ T-cells, TCRαβ^+^CD4^−^CD8^−^ T-cells, IgM^+^ plasmablasts, IgG^+^ plasmablasts, and IgA^+^ plasmablasts, the age groups of 10–17 years and >60 years contained only *n* = 18 and *n* = 21 individuals, respectively. The original data set with the age-related reference values will be available via the EuroFlow website (www.EuroFlow.org) and will continuously be updated when more data become available, also for other leukocyte subsets.

Here we describe newly designed, fully validated EuroFlow procedures and tools for comprehensive immunophenotypic diagnostic screening and classification of PID of the lymphoid system. The proposed EuroFlow PID approach relies on: 1. Optimized and validated ≥8-color antibody panels; 2. Standardized procedures for sample preparation, immunostaining, acquisition, and analysis of up to millions of cells per sample; 3. Automated gating procedures for reproducible identification of the many different immune cell subsets in blood and bone marrow (BM). A diagnostic algorithm and age-related reference values are provided for guiding the flowcytometric PID diagnosis and classification process; the entries in the EuroFlow diagnostic algorithm are based on available clinical information and basic laboratory data (Figure 1A), followed by stepwise application of the newly designed antibody combinations with age-related reference values of lymphocyte subsets in absolute counts (Figures 1A,B).

This report describes the overall EuroFlow PID approach, while detailed validation and reference value studies, including healthy subjects and PID patient series, are provided per PID tube (set) in separate EuroFlow PID reports (56–60).

## Methods

### Design of the EuroFlow-PID Study

The design of the EuroFlow PID study took advantage of the experience built in the field of leukemia and lymphoma diagnosis, classification, and monitoring (61–65) and the previously developed EuroFlow pre-analytical and analytical standard operating procedures (SOPs) for sample collection, transportation and staining of ≥10^6^ nucleated cells (63, 64), together with EuroFlow 8-color instrument set-up and calibration procedures (62), extended to ≥12-color flow cytometry (56). Multicenter evaluation of the performance of antibody panels was done in consecutive cycles of design-testing-evaluation-redesign in large series of healthy controls and patient samples in 10 EuroFlow centers, experienced in PID diagnostics (56–59). For this purpose we used EuroFlow multivariate analytical tools (66), incorporated in the Infinicyt software and developed by Cytognos SL (Salamanca, Spain).

Stepwise application of newly-designed and validated antibody combinations and available clinical and laboratory information resulted in an algorithm for guiding immunophenotypic diagnosis and classification of PID. The final versions of the EuroFlow PID tubes were used to build EuroFlow databases of normal and patient samples, for automated classification of cell populations (i.e., automated gating) and disease profiles (i.e., orientation of PID diagnosis and classification), as described in detail elsewhere (64, 65, 67).

The multiple cycles of design-testing-evaluation-redesign started in 2012 and took a total of 6 years and 20 in-person EuroFlow PID meetings to reach the final results. No single EuroFlow laboratory could have afforded the above described efforts on its own. Solely thanks to intensive collaboration and frequent exchange of results and information during the EuroFlow meetings, the here described results could be achieved, supported by local funds and by royalty income from pre-existing EuroFlow patents in the leukemia-lymphoma field.

### Flow Cytometers and Instrument Settings and Calibration

Most laboratories (9 out of 10) used FACSCanto-II flowcytometers (BD Biosciences, San Jose, CA), one laboratory used a Navios flowcytometer (Beckman-Coulter, Hialeah, FL). Standardized EuroFlow SOPs for instrument set-up and calibration were used for both instruments, as provided in detail via the EuroFlow website (www.EuroFlow.org) and by Kalina et al. (62). With such protocols, fully comparable results are obtained as previously demonstrated for both FACSCanto II, Navios, and other ≥8-color instruments, even when run by different operators (68).

For condensing sets of two 8-color tubes into single 12-color tubes, BD LSR Fortessa X-20 or FACSLyric instruments (BD Biosciences) were used in four centers where the EuroFlow instrument set-up and calibration SOPs were extended for the required extra colors, as described elsewhere (56, 58) and on the EuroFlow website (www.EuroFlow.org).

### EuroFlow Standard Operating Procedures for Sample Preparation and Acquisition of High Cell Numbers

To gain detailed insight into the composition of the lymphocyte compartment, including robust identification of small cell populations such as plasmablasts subsets, EuroFlow developed SOPs for acquisition of high cell numbers (≥1–5 × 10^6^ total nucleated cells) and/or large sample volumes (up to 2 mL per tube) (56, 63, 64). These procedures can be used for fresh (<36 h, preferably <24 h) blood and BM samples (69).

For acquisition of high cell numbers, the EuroFlow bulk-lysis-and-stain technique is recommended (56, 64), as described also on the EuroFlow website (www.EuroFlow.org). Briefly, the sample (up to 2 mL) is diluted in a total volume of 50 mL of an ammonium chloride hypotonic solution (1:25 mL vol:vol per 50 mL tube), gently mixed and incubated for 15 min in a roller. Then, nucleated cells were centrifuged and washed twice in phosphate buffered saline (PBS) containing 0.5% bovine serum albumin (BSA). Subsequently, the surface membrane markers on nucleated cells are stained with the corresponding antibody mixtures. Overall, ≥1–5 × 10^6^ nucleated cells were measured for each antibody combination.

### Construction of Antibody Panels

Antibody panels were designed for unequivocal identification and full dissection of lymphocyte subsets and their maturation-associated pathways, in parallel to other leukocyte subpopulations, which might show uniquely altered patterns in different PID categories. Specific combinations of fluorochrome-conjugated reagents were selected based on the need for brightness, stability, limited fluorescence spill-over and compensation requirements, as described elsewhere (58, 62, 70). These antibody combinations were evaluated in parallel in multiple centers (at least 4 centers per testing round) and they were optimized via multiple consecutive cycles of design-testing-evaluation-redesign. In each testing cycle, the Infinicyt software was used to identify antibodies for optimal recognition and clear-cut separation of the target cell subsets, while other antibodies were discarded because of insufficient separation of target cell subsets, poor contribution and/or redundancy, as exemplified for the PIDOT in Van der Burg et al. (59). Once optimal recognition and separation of the different target cell subsets was achieved with high reproducibility among the different laboratories, the antibody combination was frozen for final validation in large series of normal and PID patient samples (56, 59, 60). This included an intra-laboratory and inter-laboratory coefficient of variation (CV) for the identification of different minor and major lymphoid subsets of <10 and <30%, respectively, as described for example for the PIDOT (60) and the IgH-isotype tube (57).

Optimal recognition and clear-cut separation of target cell populations avoids arbitrary marker settings between cell populations with vague cutoff values, which easily vary between different laboratories, particularly when different antibody clones and fluorochrome conjugates are used. Therefore, the above described procedures are essential for obtaining reproducible results, which allow comparison of flowcytometric patterns of PID patients between centers at the international level.

### Patients and Age-Matched Healthy Controls

The proposed antibody panels were extensively evaluated in multicenter studies by analyzing blood (*n* = 541) and BM (*n* = 43) samples. Blood samples from healthy controls (*n* = 300) included different age groups: cord blood (*n* = 15), newborns (*n* = 16), 1–11 months (*n* = 19), 12–23 months (*n* = 30), 2–4 years (*n* = 35), 5–9 years (*n* = 28), 10–17 years (*n* = 33), 18–60 years (*n* = 79), and >60 years (*n* = 66).

The PID patients included in the study were all genetically-defined cases, except for the most frequent subgroups of predominantly antibody deficiencies (CVID, Ig-subclass deficiencies), for which the ESID and IUIS diagnostic and classification criteria (1, 5) were applied. The PID patients (*n* = 241) concerned: SCID (*n* = 24), CVID (*n* = 66), DiGeorge syndrome (*n* = 6), ALPS (*n* = 5), Wiskott-Aldrich Syndrome (*n* = 3), selective IgA-deficiency (*n* = 68), BTK-deficiency (*n* = 10), CD40L-deficiency (*n* = 6), other less profound CID (*n* = 6), other CID with syndromic features (*n* = 11), and several other PID subgroups (*n* = 36), such as IgG subclass deficiency with IgA deficiency (*n* = 10), PI3K delta syndrome (*n* = 5), GATA2 deficiency (*n* = 5), other diseases of immune dysregulation (*n* = 4), together with other PID patients not classified as primary defects of the lymphoid system, e.g., chronic granulomatous disease (*n* = 5), defects of innate immunity (*n* = 3) and complement deficiencies (*n* = 4) (59).

Normal blood samples were obtained from healthy adult volunteers or from children upon informed consent of the parents. The BM samples concerned remaining cell material of sibling BM stem cell transplantation donors who consented to participate in the study. All normal and patient samples were collected in tubes containing EDTA as anti-coagulant and processed within 24 h after sampling (69).

Ethical approval and informed consent procedures were according to the local ethical guidelines of the participating EuroFlow institutions and the Declaration of Helsinki (University of Salamanca, Salamanca, Spain; Charles University, Prague, Czech Republic; La Paz Hospital, Madrid, Spain; Erasmus MC, Rotterdam, The Netherlands; University Hospital Ghent, Belgium; and St. Anne's University, Brno, Czech Republic). The study was approved by the local ethics committees of the participating centers: University of Salamanca, Salamanca, Spain (USAL CSIC 20-02-2013); Charles University, Prague, Czech Republic (15-28541A); Erasmus MC, Rotterdam, The Netherlands (MEC-2013-026); University Hospital Ghent, Belgium (B670201523515); and St. Anne's University, Brno, Czech Republic (METC 1G2015).

### Data Acquisition and Data Analysis With EuroFlow Software Tools

Data acquisition was performed at low-medium speed (5,000–10,000 cells/s) using either FACSDiva (version 8) or the Navios software. For data analysis, the Infinicyt software (version 2.0) was used. Briefly, standardized Boolean gating strategies were defined and used for manual gating of the distinct cell populations identified in each tube. The merge function of the Infinicyt software was used to merge data files into reference databases. For each cell population its relative distribution among all nucleated cells, lymphocytes, and the corresponding B-, T-, and NK-cell subsets, were calculated and the MFI values per marker reported for each cell subset identified. In addition, absolute lymphocyte counts were calculated using a CD45PerCP, CD3FITC, CD19APC, and CD16^+^CD56PE TruCOUNT tube (BD Biosciences) following the instructions of the manufacturer.

For automated identification of the cell populations present in the PID tubes, PID databases were built, containing normal blood samples stained with the same antibody combination(s). Reference ranges with abnormality alarms were set per age-group, based on the analysis of a large cohort of 250 healthy control samples: cord blood (*n* = 15), childhood <18 years (*n* = 146), and adults ≥18 years (*n* = 89).

### Multicenter Validation of PID Tubes

In order to ensure full comparability between the MFI per marker per cell subset in different samples stained at distinct centers, all EuroFlow centers were trained in the EuroFlow instrument set-up and calibration as well as sample preparation SOPs. Afterward, each center was enrolled in the EuroFlow Quality Assurance program (71, 72). EuroFlow QA program showed that overall QA results of EuroFlow laboratories showed CVs below 30% in more than 90 and 70% of cell populations in 59/72 (82%) and in 71/72 (99%) QA sets, irrespectively of the flowcytometer used and the participant.

In addition, normal blood samples stained with the PID Orientation tube upon strictly following the EuroFlow SOPs, showed ≤ 1% abnormality alarms for the normal lymphocyte and myeloid cell populations.

## Results

### EuroFlow Algorithm for PID Diagnosis and Classification

Suspicious patient features, such as recurrent and unusual infections, particularly with the same pathogen, auto-immunity, inflammation, lymphoproliferation, and/or organomegaly are triggers for application of the “PID Orientation tube” (PIDOT). In line with the EuroFlow PID algorithm (Figure 1A), the results of the PIDOT will guide the next steps:

- When the PIDOT identifies s*trongly reduced B-cell counts* (<1 cell/μL; see Figure 1B) in the absence of a T-cell problem, the diagnosis of agammaglobulinemia is likely. In such case analysis of the B-cell precursor (BCP) compartment in BM with the PID-BCP tube might be informative to define the position and degree of blockade in early B-cell development, which differs between different genetic defects (37, 38).
- In case the PIDOT reveals *reduced B-cell (subset) counts* (<5th percentile ≈ <2 SD values; see Figure 1B), application of the “Pre- and Post-GC B-cell tubes” is advised.
- The results of the “Pre- and Post-GC B-cell tubes” might directly support the diagnosis of Hyper IgM syndrome or CD19 complex deficiency (Figure 1A).
- If the “Pre- and Post-GC B-cell tubes” do not detect plasmablasts in blood (<0.01 cell/μL; see Figure 1B), in the presence of reduced memory B-cell subsets, the CVID diagnosis should be considered (57). The IgH isotype tube can further support such diagnosis (57).
- If the “Pre- and Post-GC B-cell tubes” reveal IgH class aberrancies in memory B-cell subsets or plasmablast subsets (<5th percentile ≈ <2 SD values; see Figure 1B), application of the IgH isotype tubes is advised.
- When the PIDOT tube identifies *virtually no (naïve) T-cells* (<1 cell/μL; see Figure 1B), application of the “SCID/recent thymic emigrant (RTE)” tube is advised to confirm the lack of T-cell production. It should be noted that SCID patients might have normal or reduced T-cell counts in the virtual absence of naïve T-cells (<1 cell/μL; see Figure 1B) with T-cell “right shifts” to more mature T-cell subsets; this is typically seen in subgroups of SCID patients such as “leaky SCID” and Omen syndrome (59) and generally appear to concern oligoclonal expansions of mature T-cells (73, 74).
- In case of *reduced T-cell (subset) counts* (<5th percentile ≈ <2 SD values; see Figure 1B), application of the T-cell subset tube is proposed.

The combined results of all (*n* = 241) evaluated PID patients demonstrated that in *patients with suspicion of predominantly antibody deficiency, e.g., recurrent respiratory infections, low serum Ig levels, and poor antibody response to vaccination*, the PID Orientation tube should (at diagnostic screening) directly be combined with the “*IgH-isotype B-cell tubes*”.

Similarly, in case of *infants with failure to thrive and severe recurrent infections with unusual pathogens*, the “SCID/RTE tube” should directly be combined with the PID Orientation tube at diagnostic screening. Such combined SCID/RTE + PIDOT tube approach will also be useful for positive cases from newborn screening (NBS) programs, i.e., cases with strongly reduced T-cell receptor excision circles (TRECs) in blood (14, 75–77).Whenever *specific diseases such as ALPS or XLP are suspected* (e.g., increased CD4^−^/CD8^−^ counts or increased total T-cells counts; Figure 1B) additional studies need to be performed (not addressed in this manuscript).

### PID Orientation Tube

**Figure 2:**
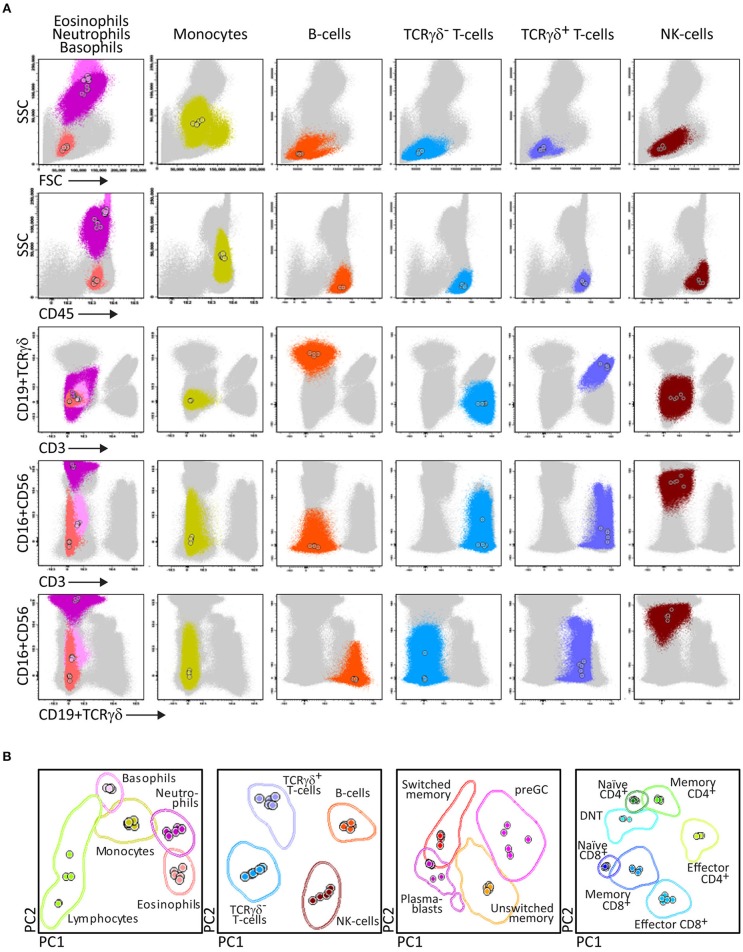
Generation of a reference principal component analysis 1 (PCA1) vs. PCA2 representation in an n-dimensional space for discrimination of the lymphocytes subsets identified with PIDOT. **(A)** Data files from 5 healthy donors were merged and lymphocyte subsets of interest identified using bivariate plots. **(B)** The merged data were used to define an n-dimensional space with the best principal component analysis 1 (PC1) vs. PC2 representation to discriminate these subsets. The PCA representation of the data is resumed in 2 × Standard Deviation (SD) curves to be used as a reference for supervised automatic analysis of the samples.

The PID Orientation tube has been designed for full dissection of all major blood leukocyte (sub)populations (*n* = 27) in a single tube (Figure 2). The choice of markers, corresponding antibodies and fluorochromes aim at reliable detection and quantitation of these blood leukocyte subsets and their potential alterations.

After four cycles of design-testing-evaluation-redesign, the final version of the 8-color PID Orientation tube consisted of 12 markers: CD27, CD45RA, CD8, IgD, CD16, CD56, CD4, IgM, CD19, CD3, CD45, and either TCRαβ or preferably TCRγδ, wherein the following antibody pairs CD8/IgD, CD16/CD56, CD4/IgM, and CD19/TCRγδ or CD19/TCRαβ are conjugated to the same fluorochrome (Table 1). These reagents aim at detailed dissection of eight major blood leukocyte subsets (Figure 2): B-cells, T-cells, NK-cells, monocytes (including non-classical CD16^+^ monocytes), dendritic cells, basophils, neutrophils and eosinophils. Additionally, B-cells and T-cells can be classified into a total of four and twelve different maturation pathway-associated subsets, respectively. Accordingly, B-cells are divided into pre-GC B-cells (including both immature/transitional and naïve B-cells), unswitched (including IgM^+^IgD^+^, IgM-only and IgD-only) and Ig-switched memory B-cells (MBC) and generally also plasmablasts (Figure 2). The T-cell compartment can be divided into different functional subsets according to the TCRγδ or TCRαβ lineage and according to CD4 and CD8 expression. These T-cell subsets can be further subdivided according to their maturation stage into naïve, central/transitional memory, and effector memory/terminally differentiated T-cells (Figure 2).

Table 1Composition of the 8-color PID Orientation tube and technical information on reagents.

**Table:** 

**BV421**	**BV510**	**FITC**	**PE**	**PerCPCy5.5**	**PECy7**	**APC**	**APCH7**
CD27	CD45RA	CD8 and	CD16 and	CD4 and	CD19 and	CD3	CD45
		SmIgD	CD56	SmIgM	TCRγδ

**Table:** 

**Marker**	**Fluorochrome**	**Clone**	**Source**	**Catalog number**	**Application in EuroFlow panel**	**μl/test**
CD3	APC	SK7	BD Biosciences	345767	Orientation, SCID/RTE, T cell subset tubes	2.5
CD4	PerCPCy5.5	SK3	BD Biosciences	332772	Orientation tube	7
CD8	FITC	SK1	BD Biosciences	345772	Orientation tube	5
CD16	PE	3G8	BD Biosciences	555407	Orientation tube	5
CD19	PECy7	J3-119	Beckman Coulter	IM3628	Orientation, BM-BCP, Pre-GC, Post-GC, IgH-isotype tubes	5
CD27	BV421	M-T271	BD Biosciences	562513	Orientation, Pre-GC, Post-GC, T cell subset tubes	1
CD27^*^	*BV421*	*O323*	*BioLegend*	*302824*	Orientation, Pre-GC, Post-GC, T cell subset tubes	1
CD45	APCH7	2D1	BD Biosciences	641417	Orientation tube	2
CD45RA	BV510	HI100	BD Biosciences	563031	Orientation tube	2.5
CD45RA^*^	*BV510*	*HI100*	*BioLegend*	*304142*	Orientation tube	2.5
CD56	PE	C5.9	Cytognos	CYT-56PE	Orientation tube	5
CD56^*^	*PE*	*Leu11c*	*BD Biosciences*	*332779*	Orientation tube	5
SmIgD	FITC	IA6-2	BioLegend	348205	Orientation tube	1.25
SmIgM	PerCPCy5.5	MHM-88	BioLegend	314511	Orientation tube	2
TCRγδ	PECy7	11F2	BD Biosciences	655410	Orientation, SCID/RTE, T cell subset tubes	1

* *Alternative reagents tested to provide same results*.

**Figure 3:**
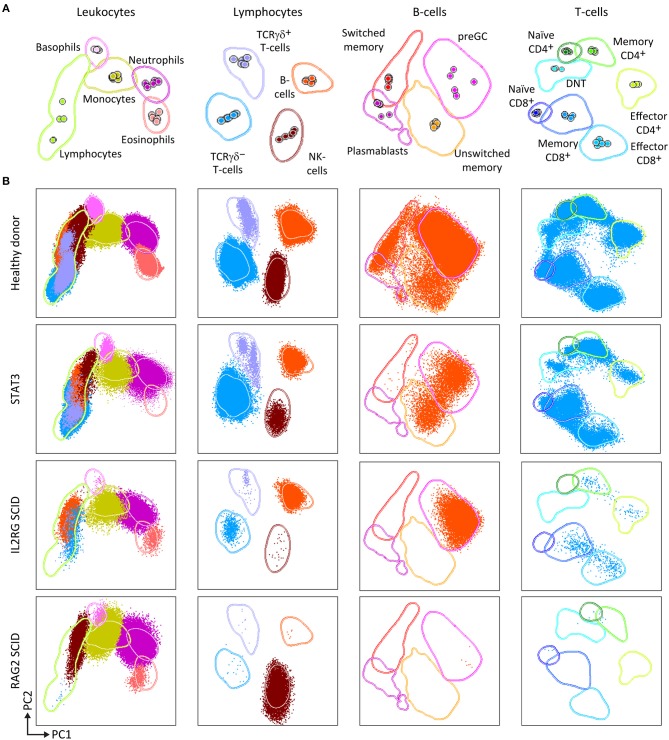
PCA representation of PIDOT results, showing the distinct blood leukocyte subsets in the reference data file and supervised analysis of blood samples from a healthy donor and three different PID patients. **(A)** Reference principal component analysis. PCA1 vs. PCA2 representation was generated from five healthy donors data files, analyzed with the EuroFlow PIDOT. **(B)** Different patient samples were analyzed against the healthy donor PCA reference. Blood samples from a healthy donor, a patient with mutated STAT3, a SCID patient with IL2 receptor gamma chain (IL2RG) deficiency, and a SCID patient with recombination activating gene 2 (RAG2) deficiency were stained and acquired under identical/comparable conditions. The STAT3 deficient patient shows the typical pattern of reduced naïve T-cells, virtually no IgH-class switched B-cells, and no eosinophils. The IL2RG deficient SCID patient has virtually no T-cells, particularly no naïve T-cells (<1 cell/μL; see Figure 1B), B-cells are present, but no memory B-cells or plasmablasts, and NK-cells are virtually absent. The RAG2 deficient SCID patient has no B-cells and no T-cells, while NK-cells are present in normal numbers. DNT, double negative T-cells, negative for CD4 and CD8, but positive for CD3.

Therefore, the PID Orientation tube detects defects in the production of B-cells, T-cells and NK-cells, together with alterations (defective but also increase) in the production of monocytes, dendritic cells, neutrophils, eosinophils, and basophils. Three typical PID patient examples (STAT3, IL2RG, and RAG2 deficiency) are shown in Figure 3 to illustrate the disease-associated immunophenotypic profiles. However, in most cases the PID Orientation tube does not allow precise (sub)classification of the T-cell and B-cell defects, implying that further characterization is required with additional B-cell and T-cell tubes, according to the EuroFlow PID algorithm (Figure 1A), as described below [see also (59)].

Some diagnostic laboratories in the PID field use a simple B-T-NK tube as first screening step, while most laboratories use several 4- to 6-color tubes in parallel to perform initial screening (generally using 12–18 antibodies with multiple repeats). The here proposed 8-color tube with 12 antibodies provides information on up to 20 different leukocyte (sub)populations, clearly separated in multidimensional principal component analysis (Figures 2, 3), thereby providing the basis for further classification of PID of the lymphoid system.

### B-Cell Tubes

For antibody deficiencies and combined T/B-cell defects, detailed analysis of the B-cell compartment in blood and BM is required. B-cells originate from the BCP differentiation pathway in BM into immature and naïve B-cells that enter the periphery, including blood. In the periphery, the B-cells encounter antigen which induces a GC reaction. Consequently, blood contains naïve B-cells that did not yet undergo a GC reaction (pre-GC B-cells) or that have already been exposed to antigen, such as antigen-experienced memory B-cells (MBC), plasmablasts and plasma cells. The antigen-experienced subsets can be further subdivided according to class switching of their IgH-isotypes (and subclasses): IgM, IgD, IgE, IgG (including IgG1, IgG2, IgG3, IgG4), and IgA (including IgA1, IgA2).

**Figure 4:**
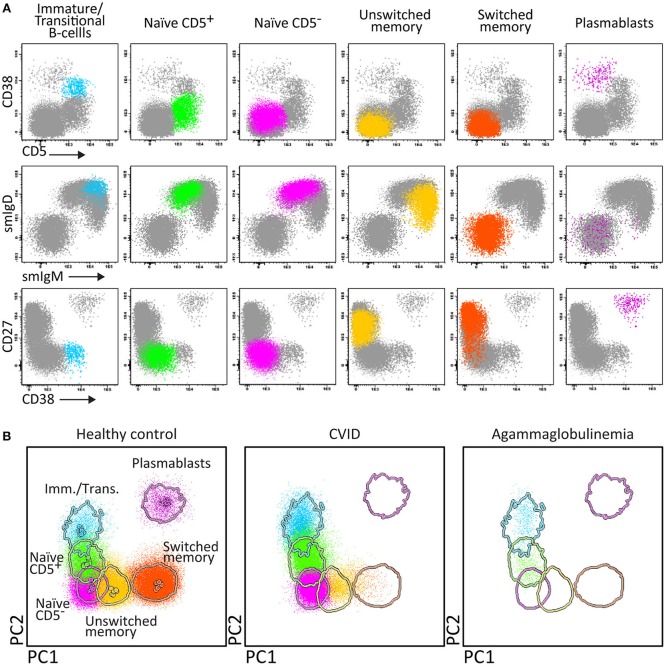
Application of the Pre-GC tube, showing the major B-cell subsets in blood of healthy controls, and PID patients using classical 2-dimensional plots vs. n-dimensional PCA. Analysis of circulating B-cell subsets (FS/SS^lo^CD19^+^) identified within 2 × 10^6^ blood leukocytes using the markers from the Pre-GC tube: Immature (CD5^+^ CD27^−^ CD38^++^ smIgM^++^ smIgD^+^), CD5^+^ and CD5^−^ naïve B-cells (CD27^−^ CD38^+d^ smIgM^+^ smIgD^++^), Ig non-switched and Ig-switched memory B-cells (CD5^−^ CD27^+^ CD38^−^ smIgM^+^ smIgD^++^ and CD5^−^ CD27^+/−^ CD38^−^ smIgM^−^ smIgD^−^, respectively), and plasmablasts (CD5^−^ CD27^++^ CD38^+++^). **(A)** The identification of biologically relevant subsets of B-cells using the minimum number of bivariate plots required for a 5 marker-combination (three bivariate plots/cell population). **(B)** The same data via PC1 vs. PC2 representation of a 5-dimensional space in blood of six healthy controls (left panel), in blood of a patient with common variable immunodeficiency (CVID; middle) and in blood of a patient with agammaglobulinemia (right), all stained with the Pre-GC tube under comparable conditions. The CVID patient lacks plasmablasts in blood (<0.01 cell/μL blood) and has reduced IgH-class switched MBC. The agammaglobulinemia patient has no plasmablasts, no MBC, and also the naïve B-cell compartment is strongly reduced.

Therefore two 8-color panels were designed for the identification of (i) different pre-GC B-cell subsets, including immature/transitional, naïve CD5^+^ and naïve CD5^−^ B-cells, and (ii) antigen-experienced subsets, including MBC and plasmablasts (Figure 4). The latter panel includes sub-classification of both MBC and PC according to IgH-isotype (IgM, IgD, IgG, IgA, IgE). Further IgH subsetting according to the specific subclasses (IgM, IgD, IgG1, IgG2, IgG3, IgG4, IgA1, and IgA2) was designed in a combination of two separate 8-color tubes or a single 10 or 12-color tube (56).

Accordingly, the EuroFlow 8-color PID B-cell tube set consists of five tubes for detailed subsetting of blood pre-GC B-cells (Pre-GC B-cell tube), antigen-experienced B-cells (Post-GC B-cell tube), including detailed IgH-isotype and subclass analysis of MBC and plasmablasts (IgH-isotype B-cell tubes 1 and 2), and BM BCP analysis (BCP tube) (Figure 1A).

**Figure 5:**
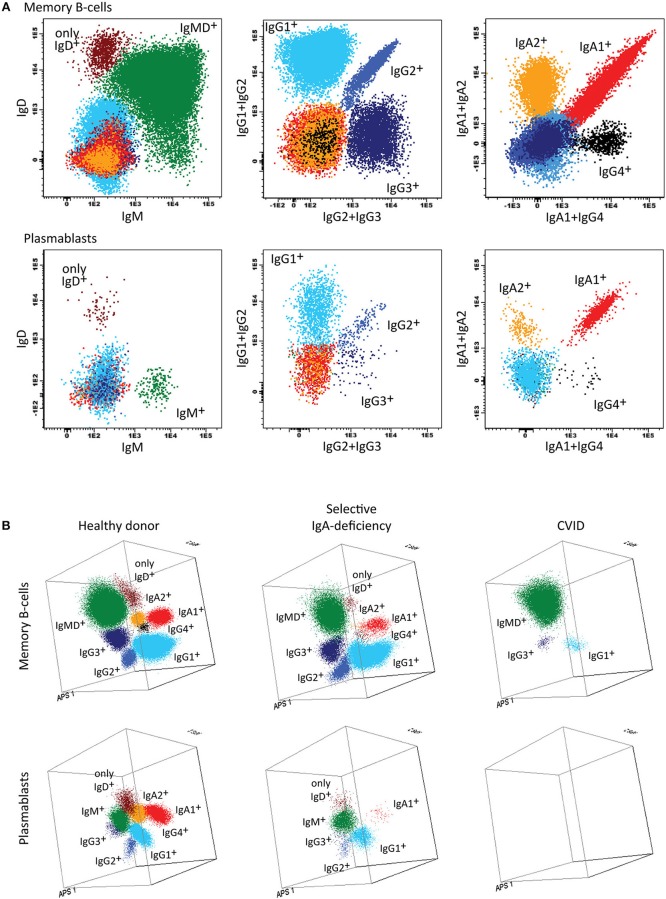
Application of the IgH-isotype tube for the dissection of switched memory B-cells and plasmablasts in blood from healthy donors and PID patients. **(A)** Bivariate plots showing the distribution of switched memory B-cells (smIgMD^−^CD19^+^CD38^−^) and plasmablasts (CD19^+^CD27^++^CD38^+++^) according to the surface membrane expression of the different IgH-isotypes (IgG1, IgG2, IgG3, IgG4, IgA1, and IgA2) in 5 × 10^6^ peripheral blood leukocytes analyzed with the IgH-isotype tubes in samples from a healthy adult. **(B)** Three dimensional PCA representation of IgH-isotype subsets in memory B-cells (top) and plasmablasts (lower) in a healthy adult donor (left), a selective IgA-deficient patient (middle), and a CVID patient (right). No plasmablasts were detected in blood from the CVID patient (count of <0.01 cells/μL blood), while variable defects were detected in the memory B-cell compartment, particularly involving switched memory B-cells. The defects in the IgA-deficient patient mainly concerned the IgA-class-switched plasmablasts.

Briefly, the **“Pre-GC B-cell tube”** contains CD27, IgM, CD38, CD5, IgD, CD19, CD21, and CD24 markers (Table 2), which allows detection of immature/transitional B-cells, CD5^+^ and CD5^−^ naïve B-cells (including their CD21 and CD24 subsets), unswitched (including IgM^+^IgD^+^, IgM-only, and IgD-only) and switched MBC and PC. The **“Post-GC B-cell tube”** contains CD27, IgM, IgA, IgG, IgD, CD19, CD21, CD38, and optionally IgE, wherein two distinctly labeled antibodies against IgA allow to have the IgE/IgA pair and IgG/IgA pair conjugated to the same fluorochrome (Table 2) (78). The **“IgH-Isotype B-cell tube-1 and tube-2”** (Figure 5A) both contain CD27, IgM, IgD, CD19, CD21, and CD38 together with IgG4, IgG2, IgG1, or with IgA2, IgA1, IgG3, respectively. In this composition two distinctly labeled antibodies against IgG2 and IgA1 are used, wherein the antibodies within the pairs IgG1/IgG2 and IgG2/IgG4 or IgA1/IgG3 and IgA1/IgA2 are conjugated to the same fluorochrome (Table 2).

Table 2Composition of the 8-color B-cell tubes and technical information on reagents.

**Table:** 

	**BV421**	**BV510**	**FITC**	**PE**	**PerCPCy5.5**	**PECy7**	**APC**	**APCAF750**
Pre-GC	CD27	SmIgM	CD38	CD5	SmIgD	CD19	CD21	CD24
Post-GC	CD27	SmIgM	SmIgE and SmIgA	SmIgG and SmIgA	SmIgD	CD19	CD21	CD38
IgH-isotype-I	CD27	SmIgM	SmIgG4 and SmIgG2	SmIgG1 and SmIgG2	SmIgD	CD19	CD21	CD38
IgH-isotype-II	CD27	SmIgM	SmIgG3 and SmIgA1	SmIgA2 and SmIgA1	SmIgD	CD19	CD21	CD38

**Table:** 

**Marker**	**Fluorochrome**	**Clone**	**Source**	**Catalog number**	**Application in EuroFlow-PID panel**	**μl/test**
CD5	PE	UCHT-2	BioLegend	300608	Pre-GC tube	5
CD19	PECy7	J3-119	Beckman Coulter	IM3628	Orientation, BM-BCP, Pre-GC, Post-GC, IgH-isotype tubes	5
CD21	APC	B-ly4	BD Biosciences	559867	Pre-GC, Post-GC, IgH-isotype tubes	10
CD24	APCAF750	ALB9	Beckman Coulter	B10738	Pre-GC,	5
CD27	BV421	M-T271	BD Biosciences	562513	Orientation, Pre-GC, Post-GC, IgH-isotype, T-cell subset tubes	1 (Pre/Post-GC)/2 (IgH-isotype)
CD38	FITC	HB7	BD Biosciences	340909	Pre-GC tube	5
CD38	APCH7	HB7	BD Biosciences	656646	Post-GC tube, IgH-isotype	3
SmIgA	FITC	IS11-8E10	Miltenyi	130-093-071	Post-GC tube	1
SmIgA	PE	IS11-8E10	Miltenyi	130-093-128	Post-GC tube	1
SmIgA1	FITC	SAA1	Cytognos	CYT-IGA1F	IgH-isotype tube	3
SmIgA1	PE	SAA1	Cytognos	CYT-IGA1PE	IgH-isotype tube	3
SmIgA2	PE	SAA2	Cytognos	CYT-IGA2PE	IgH-isotype tube	3
SmIgD	PerCPCy5.5	IA6-2	BioLegend	348208	Pre-GC, Post-GC, IgH-isotype tubes	1.5
SmIgE	FITC	polyclonal	Life Technologies	H15701	Post-GC tube	2
SmIgG	PE	G18-145	BD Biosciences	555787	Post-GC tube	20
SmIgG1	PE	SAG1	Cytognos	CYT-IGG1PE	IgH-isotype tube	3
SmIgG2	FITC	SAG2	Cytognos	CYT-IGG2F	IgH-isotype tube	3
SmIgG2	PE	SAG2	Cytognos	CYT-IGG2F	IgH-isotype tube	3
SmIgG3	FITC	SAG3	Cytognos	CYT-IGG3F	IgH-Isotype tube	3
SmIgG4	FITC	SAG4	Cytognos	CYT-IGG4F	IgH-isotype tube	3
SmIgM	BV510	MHM-88	BioLegend	314521	Pre-G, Post-GC, IgH-isotype tubes	1.3 (Pre/Post-GC)/2 (IgH-isotype)

Finally, the **BCP-BM tube** consists of the seven cell surface membrane markers CD20, IgM, CD38, IgD, CD19, CD34, and CD10 and the three intracellular markers cyIgμ, nuTdT, and cyCD79a (Table 3). This combination of markers allows detailed analysis of the BM-BCP compartment from the CD19-negative stage until the naïve B-cells and can visualize complete and partial blocks in BCP differentiation as well as aberrant expression profiles (Wentink et al., unpublished results).

Table 3Composition of the 10-color BM-BCP tube and technical information on reagents.

**Table:** 

**PacB**	**BV510**	**BV605**	**FITC**	**PE**	**PECF594**	**PerCPCy5.5**	**PECY7**	**APC**	**APCC750**
CD20	SmIgM	CD38	nuTdT	CyCD79a	SmIgD	CyIgM	CD19	CD34	CD10

**Table:** 

**Marker**	**Fluorochrome**	**Clone**	**Source**	**Catalog number**	**Application in EuroFlow-PID panel**	**μl/test**
CD10	APCC750	HI10a	Cytognos	CYT-10AC750	BM-BCP	3
CD19	PECy7	J3-119	Beckman Coulter	IM3628	Orientation, BM-BCP, Pre-GC, Post-GC, IgH-isotype tubes	5
CD20	PacB	2H7	Biolegend	302320	BM-BCP tube	1
CD34	APC	8G12	BD Biosciences	345804	BM-BCP tube	5
CD38	BV605	HIT2	Biolegend	303532	BM-BCP tube	
CyCD79a	PE	HM57	Dako	R7159	BM-BCP tube	3
SmIgD	PECF594	IA6-2	BD Biosciences	562540	BM-BCP tube	5
SmIgM	BV510	MHM-88	BioLegend	314521	BM-BCP, Pre-GC, Post-GC, IgH-isotype tubes	2
CyIgM	PerCPCy5.5	MHM-88	BioLegend	314512	BM-BCP tube	2
nuTdT	FITC	HT-6	Dako	F7139	BM-BCP tube	10

For reasons of efficiency, the two IgH-isotype tubes can be combined into a single 10-color IgH-isotype tube with CD27, IgM, IgG3, IgG2, IgG1, IgD, CD19, CD21, CD38, IgA1, IgA2, and IgG4, with two distinctly labeled antibodies against IgG2 and two distinctly labeled antibodies against IgA1 (“10-color IgH-isotype tube”). Preferably, the four blood B-cell tubes (Pre-GC, Post-GC, and the two IgH-isotype tubes) can be combined into a single 12-color tube by the further addition of CD5 and CD24 (“12-color IgH-isotype B-cell tube”) (56, 57).

In summary, the Pre-GC and Post-GC tubes can visualize blockades in differentiation of transitional to mature naïve B-cells in e.g., a subset of XLA patients, and variable defects in IgH-switched MBC and plasmablasts, such as in patients with Hyper IgM syndrome, CD19 complex deficiencies, IgH class aberrancies, and CVID with almost systematic absence of plasmablasts (<0.01 cell/uL) (Figures 1B, 4B) (57). In addition, the IgH-isotype tubes can uncover more subtle defects in IgH-class switching e.g., in selective IgA- and IgG-subclass deficiencies (Figure 5B) (57). Finally, the BCP tube detects early blockades in BCP maturation in BM.

### T-Cell Tubes

In many cases with suspicion of PID of the lymphoid system, analysis of T-cell subsets is an essential part of making a correct diagnosis. If the PID Orientation tube indicates reduced T-cell (subset) counts or abnormal T-cell maturation, further investigation of the blood T-cell compartment is recommended. The SCID/RTE tube is meant for cases with strongly reduced (naïve) T-cell production (<1 cell/μL; see Figure 1B), whereas the T-cell subset tube should be applied when the PID orientation tubes reveals an imbalanced composition of the memory and effector T-cell compartments.

**Figure 6:**
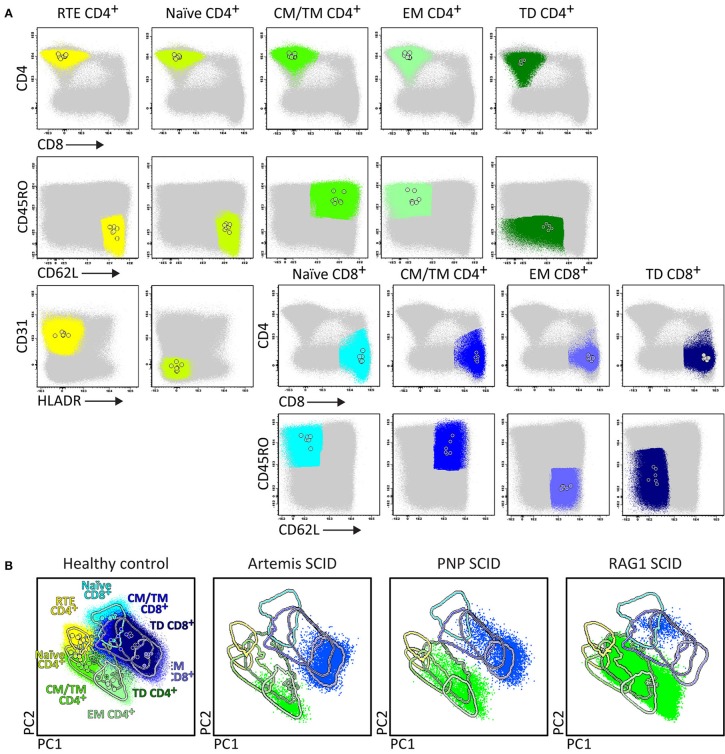
Application of the RTE-SCID tube in healthy controls and T-cell defects. Analysis of circulating T-cell subsets (FS/SS^lo^CD3^+^) identified within 1x10^6^ blood leukocytes using the markers from the RTE-SCID tube: Recent Thymic Emigrants (RTE) CD4^+^ T-cells (CD4^+^ CD8^−^ CD45RO^−^ CD62L^+^ CD31^+^ HLA-DR^−^), and naïve (CD45RO^−^ CD62L^+^ CD31^+^ HLA-DR^−^), Central/Transitional Memory (CM/TM) (CD45RO^+^ CD62L^+^), Effector Memory (EM) (CD45RO^+^ CD62L^−^), and Terminally Differentiated (TD) (CD45RO^−^ CD62L^−^) CD4^+^ and CD8^+^ T-cells. **(A)** The identification of biologically relevant T-cell subsets in six healthy controls using the minimum number of bivariate plots required for a 6 marker-combination (three bivariate plots/cell population). **(B)** Comparable T-cell data via PCA1 vs. PCA2 representation of a 6-dimensional space in six healthy controls (left) as well as in three severe combined immunodeficiency (SCID) patients, diagnosed with Artemis, PNP and RAG-1 defects, all stained with the RTE/SCID tube under comparable conditions. All three SCID patients show “leakiness” with virtually complete absence of naïve T-cells (<1 cell/μL blood) and clear “right shift” to mature CM/TM, EM, and TD T-cells in both the CD4 and CD8 lineages.

In case of high suspicion of SCID in children of <1 year with severe recurrent infections by unusual pathogens and failure to thrive, the SCID/RTE tube should be directly applied in combination with the PID Orientation tube (Figures 1A, 6). In such patients the detected blood T-cells might be (non-autologous) transplacentally-derived maternal T-cells. The predominant presence of (activated) memory T-cells (either autologous or maternal) in the absence of T-cells recently emigrated from the thymus (RTE), further supports the SCID diagnosis (Figure 6).

**Figure 7:**
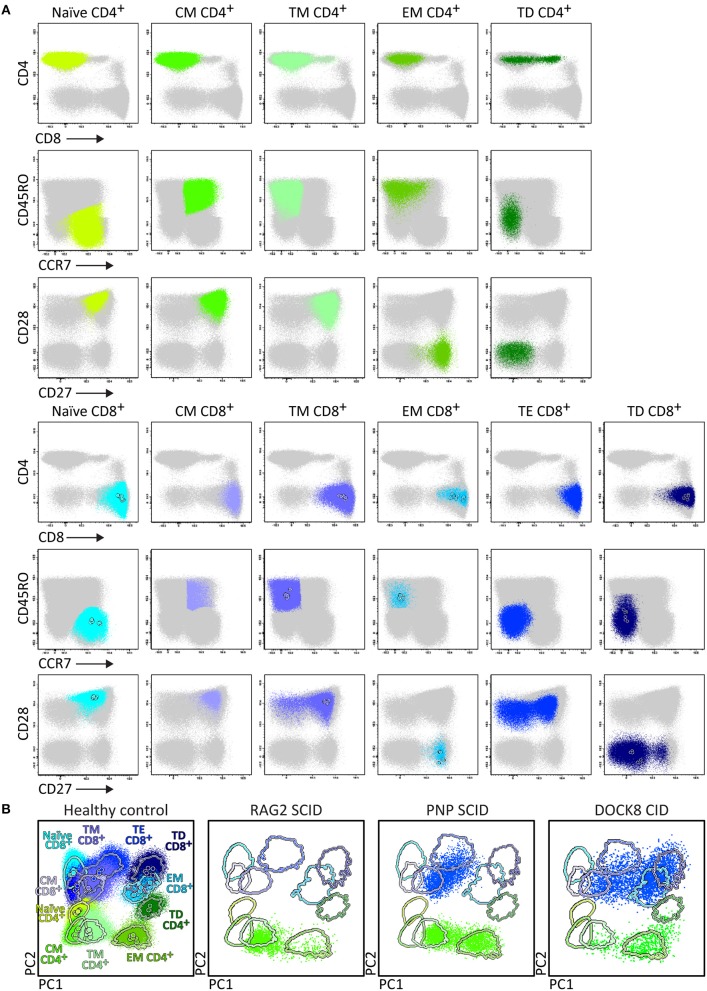
Application of the T-cell panel tube in healthy controls and PID patients with T-cell defects. Analysis of circulating T-cell subsets (FS/SS^lo^CD3^+^) identified within 1 × 10^6^ blood leukocytes using the markers from the T-cell panel tube: naïve (CD45RO^−^ CCR7^+^ CD27^+^ CD28^+^), Central Memory (CM) (CD45RO^+^ CCR7^+^ CD27^+^ CD28^+^), Transitional Memory (TM) (CD45RO^+^ CCR7^−^ CD27^+^ CD28^+/−^), Effector Memory (EM) (CD45RO^+^ CCR7^−^ CD27^−^ CD28^+^), Terminal Effector (TE) (CD45RO^−^ CCR7^−^ CD27^+^ CD28^+/−^), and Terminally Differentiated (TD) (CD45RO^−^ CCR7^−^ CD27^−^ CD28^+/−^) CD4^+^ and CD8^+^ T-cells. **(A)** The identification of biologically relevant subsets of T-cells in six healthy controls using the minimum number of bivariate plots required for a 6 marker-combination (three bivariate plots/cell population). **(B)** Comparable data via PCA1 vs. PCA2 representation of a 6-dimensional space in blood of six healthy controls (left) as compared to blood samples of three severe combined immunodeficiency (SCID) patients, diagnosed with RAG2, PNP, and DOCK8 defects, all stained with the RTE/SICD tube under comparable conditions. The SCID patients show “leakiness” with absence of naïve T-cells (<1 cell/μL blood) and variable “right shift” to mature CM/TM, EM, and TD T-cells in the CD4 lineage (all 3 SCID patients) and CD8 lineage (PNP and DOCK8 defects).

Upfront identification of the major TCRγδ, TCRαβ, CD4, and CD8 T-cell lineages is required for further detailed dissection of their maturation pathways into (CD31^+^) RTE, naïve, memory and effector subsets, including activated (HLA-DR^+^) T-cells (Figure 6). The T-cell subset tube further dissects the blood T-cell lineages into central memory (CM), transitional memory (TM), effector memory (EM), terminal differentiated (TD), and terminal effector (TE) subsets (Figure 7).

Briefly, two 8-color T-cell tubes have been designed: (i) the **SCID/RTE tube** with the five backbone markers CD3, CD4, CD8, CD45RO, and TCRαβ or TCRγδ, supplemented with the subsetting markers CD31, CD62L, HLA-DR for identification of RTEs and activated T-cells (Table 4); and (ii) the **T-cell subset tube** with the same five backbone markers, combined with CD27, CD28, and CCR7 (Table 4) for detailed dissection of the above mentioned memory and effector compartments (Figures 6, 7).

Table 4Composition of the 8-color BM-BCP tube and technical information on reagents.

**Table:** 

	**BV421**	**BV510**	**FITC**	**PE**	**PerCPCy5.5**	**PECy7**	**APC**	**APCAF750**
SCID/RTE panel	CD62L	CD4	CD45RO	CD31	HLA-DR	TCRγδ	CD3	CD8
T-cell panel	CD27	CD4	CD45RO	CD197	CD28	TCRγδ	CD3	CD8

**Table:** 

**Marker**	**Fluorochrome**	**Clone**	**Source**	**Catalog number**	**Application in EuroFlow panel**	**μl/test**
CD3	APC	SK7	BD Biosciences	345767	Orientation, SCID/RTE, T cell subset tubes	2.5
CD4	BV510	OKT4	BioLegend	317443	Orientation, SCID/RTE, T cell subset tubes	1.5
CD8	APCAF750	B9.11	Beckman Coulter	A94683	Orientation, SCID/RTE, T cell subset tubes	1.5
CD27	BV421	M-T271	BD Biosciences	562513	Orientation, Pre-GC, Post-GC, T cell subset tubes	1
CD27^*^	*BV421*	*O323*	*BioLegend*	*302824*	Orientation, Pre-GC, Post-GC, T cell subset tubes	1
CD28	PerCPCy5.5	CD28.2	BioLegend	302921	T-cell subset tube	4
CD31	PE	MEM-05	Exbio	1P-273-T100	SCID/RTE tube	5
CD45RO	FITC	UCHL1	Exbio	1F-498-T100	SCID/RTE, T cell subset tube	10
CD62L	BV421	DREG-56	Biolegend	304827	SCID/RTE tube	2
CD197	PE	FR 11-11E8	Miltenyi	130-093-621	T cell subset tube	5
HLADR	PerCPCy5.5	L243	Biolegend	307629	SCID/RTE tube	1.5
TCRγδ	PECy7	11F2	BD Biosciences	649806	Orientation, SCID/RTE, T cell subset tubes	1

* *Alternative reagent tested to provide same results*.

In summary, while the first tube identifies major defects in T-cell production, the second tube focusses on milder defects in T-cell production and/or altered T-cell responses (Figures 6, 7).

### EuroFlow Reference Databases as Essential Tool for Easy, Fast, and Reproducible Analysis of Patient Samples

Similarly to the EuroFlow leukemia-lymphoma diagnosis and monitoring databases (64–66), reference databases have been generated for the EuroFlow PID tubes to facilitate: 1. Automated gating of all leukocyte subsets; 2. Identification of PID-associated immune cell profiles vs. normal age-matched reference values and phenotypes; and 3. Further sub-classification of PID cases into distinct diagnostic categories (Figures 2–7).

**Figure 8:**
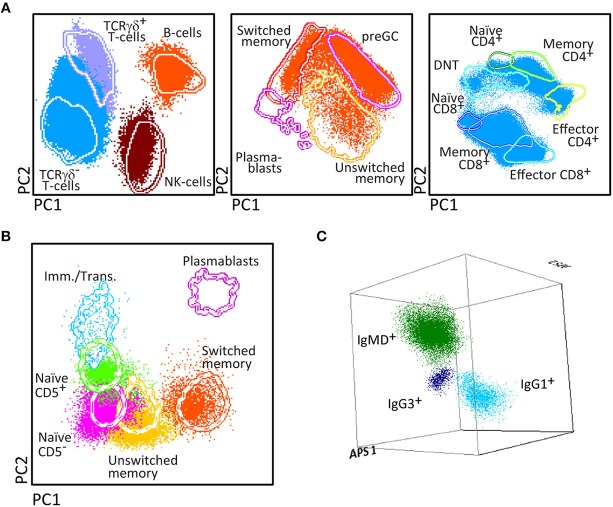
PCA representation of the biologically relevant subsets in a patient diagnosed with IgG2 subclass deficiency with IgA deficiency (40 years). The distribution of the biologically relevant PB lymphocyte subsets has been represented using multidimensional reference plots. The supervised analysis of the PID orientation tube using PC1 vs. PC2 reference plots **(A)** shows that plasmablasts were undetectable in the patient sample after acquiring 1 × 10^6^ cells. Lack of plasma cells was confirmed in the analysis of the Pre-GC and Post-GC B-cell tubes **(B)**. In addition, three dimensional PCA representation of IgH-isotype subsets in memory B-cells **(C)** showed that although the counts of total switched memory B-cells was normal in the previous tubes, the subsets of IgG2^+^, IgA1^+^, and IgA2^+^ were undetectable with a sensitivity of 10 cells in a mL of peripheral blood (0.01 cells/μL).

**Figure 9:**
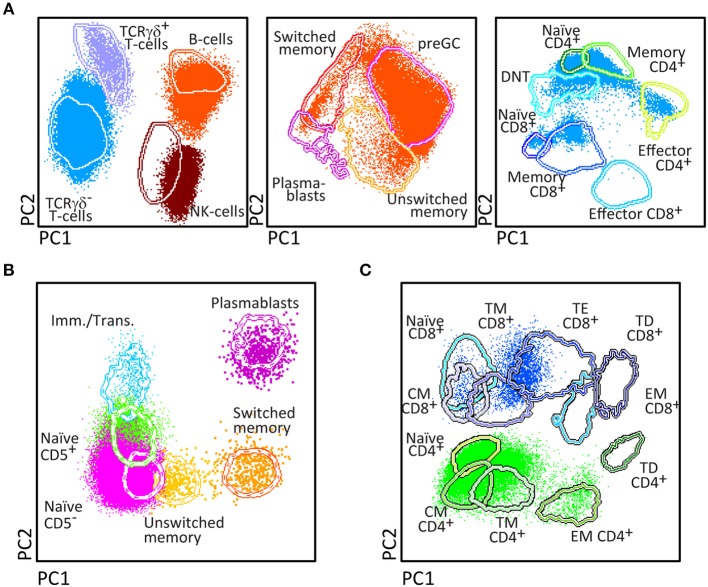
PCA representation of the biologically relevant subsets in a patient diagnosed with a WASp defect (5 years-old). The distribution of the biologically relevant PB lymphocyte subsets has been represented using multidimensional reference plots. The supervised analysis of the PID orientation tube using PC1 vs. PC2 reference plots **(A)** shows that low numbers of total and naïve CD8^+^ T-cells, and unswitched and switched memory B-cells, were detected in the patient sample after acquiring 1 × 10^6^ cells. Decreased counts of unswitched memory B-cells was confirmed in the analysis of the B-cell tubes **(B)**. In addition, PCA1 vs. PCA2 representation of the T-cell tube **(C)** confirmed that naïve CD8^+^ T-cells were decreased.

Stepwise application of the EuroFlow PID tubes according to the proposed algorithm (Figure 1A) supports the diagnosis of many PID evaluated so far (*n* = 233), and provides further classification of most lymphoid PID cases into distinct diagnostic categories according to altered flowcytometric immune cell profiles (Table 5). For example, the PIDOT together with the IgH-isotype tubes (Figure 8) support the diagnosis of virtually all IgA- and IgG-subclass deficiencies and CVID patients, including their detailed characterization (57). Figure 9 shows an example of combined application of the PIDOT, the Pre-GC tube and the T-cell subset tube in a patient with a WASp defect, showing several aberrancies in T-cell subsets.

**Table 5:** Application of EuroFlow PID tubes for the primary immunodeficiency disease categories.

**Disease category (IUIS 2017)^1^**	**EuroFlow PID tubes**						
	**Orientation**	**Pre-GC**	**Post-GC**	**IgH-isotypes**	**SCID/RTE**	**T-cell subset tube**	**Frequency correct diagnosis**
Immunodeficiency affecting cellular and humoral immunity (*n* = 36)	Diagnostic screening	Exploratory	Exploratory	Exploratory	Clinical classification	Clinical classification	100%
5CID with associated or syndromic features (*n* = 20)	Diagnostic screening	Exploratory	Exploratory	Exploratory	Clinical classification	Clinical classification	75%^*^
Predominantly antibody deficiencies (*n* = 150)	Diagnostic screening	Clinical classification	Clinical classification	Diagnostic screening and classification	Exploratory	Exploratory	100%
Diseases of immune dysregulation (*n* = 10)	Diagnostic screening	Exploratory	Exploratory	Exploratory	Exploratory	Exploratory	90%
Congenital defects of phagocyte numbers or functions (*n* = 10)	Immuno-evaluation	Exploratory	Exploratory	Exploratory	Exploratory	Exploratory	70%
Defects in intrinsic and innate immunity (*n* = 3)	Immuno-evaluation	Exploratory	Exploratory	Exploratory	Exploratory	Exploratory	67%
Autoinflammatory disorders (*n* = 0)	Immuno-evaluation	Exploratory	Exploratory	Exploratory	Exploratory	Exploratory	–
Complement deficiencies (*n* = 4)	Immuno-evaluation	Exploratory	Exploratory	Exploratory	Exploratory	Exploratory	0%

* *60% of cases showing normal blood lymphocyte subset counts were DiGeorge Syndrome patients. Diagnostic screening: mandatory for the diagnosis and management of the patient according to international classifications (1, 2, 5, 40). Clinical classification: required for identification of subgroups of patients with different disease presentation and outcome, including guiding genetic testing (1, 2, 5, 41, 43). Immuno-evaluation: provides information indicated for treatment decision and patients monitoring (11–13). Exploratory: not required for clinical management, might provide relevant immune information; CID, Combined Immunodeficiency*.

More detailed results on the application of the individual EuroFlow PID tubes and their corresponding reference databases and age-matched reference values are provided in separate publications (57, 59) and are implemented in the EuroFlow-based Infinicyt software, respectively.

## Discussion

Diagnosis and classification of rare diseases deserves well-defined strategies that are efficient and easily accessible, providing a diagnosis at an early stage, in order to prevent diagnostic delays with higher chances of irreversible organ damage. In case of PID, this requires awareness of general practitioners and pediatricians about the most efficient and cost-effective diagnostic pathways as proposed by ESID and IUIS (2, 5, 6). These diagnostic pathways include flowcytometric immunophenotyping at an early stage, which can guide other diagnostic tests such as functional assays and genetic studies.

Specialized reference centers are required for full diagnostics and clinical management of rare diseases such as PID. Broad and easy access to fast diagnostic screening strategies and linkage to these highly specialized PID reference centers of excellence shall reduce morbidity and mortality of PID patients. The medical indications for testing of PID are well-defined (“***the 10 warning signs of PID***”) and several consensus reports on stepwise diagnostic strategies have been proposed (2, 5, 7, 79, 80). However practical implementation of these consensus proposals has only partially been achieved. This is mainly due to lack of standardization in the pre-analytical and analytical laboratory procedures, particularly in flow cytometry and NGS/WES (9, 10, 43–45). At least in part, this lack of standardization is caused by the fast technological developments in both fields, which has led to great variability in technical procedures and assays between individual laboratories, thereby hampering comparability of diagnostic results, even among reference PID centers.

The main objectives of the EuroFlow Consortium were to innovate and standardize the flowcytometric techniques and strategies, applied in the diagnosis and classification of PID of the lymphoid system and to generate reliable and reproducible results across laboratories and countries, for guiding both functional testing and genetic testing in flowcytometrically-defined PID subgroups. To achieve these objectives, the EuroFlow Consortium took advantage of its concepts, technologies, tools and experience from the field of leukemia and lymphoma diagnosis, classification and monitoring (61–65). Consequently, we sequentially (i) developed 8-color tubes (and one 12-color BM tube) for efficient diagnostic testing and classification of PID in multiple multicenter cycles of design-testing-evaluation-redesign, following strict rules for selection of optimal combinations of antibody clones and their fluorochrome conjugates; (ii) validated the approved antibody tubes in healthy controls and PID patient series (56–59); (iii) constructed reference databases of normal samples and well-annotated patient samples, which can serve as templates for prospective data analysis; and (iv) provided the basis for standardized interpretation of the results obtained in individual laboratories, which apply the same EuroFlow methods and tools.

As evaluated in more than 240 PID patients, the stepwise application of the proposed tubes according to the EuroFlow PID algorithm (Figure 1A) provides efficient and cost-effective flowcytometric diagnostic screening and classification of virtually all PID of the lymphoid system, based on fast, sensitive, easy, and reproducible identification and enumeration of all relevant subsets (Figure 1). The B-cell tubes have proven to more accurately dissect the blood memory B-cell and plasmablast compartments than achieved previously, thereby providing new possibilities to better diagnose and classify antibody deficiencies, including IgH-subclass deficiencies and CVID (57). In patients suspected of SCID (e.g., in the TREC-based NBS programs), the combined application of the PIDOT and SCID tubes will be highly informative (14, 75–77). Furthermore, the diagnostic procedures for secondary immunodeficiencies might profit as well from the proposed PID tubes and tools (81). Still several subsets of PID patients might present with no or minimally altered lymphoid subset numbers, such as in some DiGeorge patients and part of ALPS, Nijmegen breakage syndrome and ataxia telangiectasia patients at young age. In such cases functional and genetic testing are more informative. Finally, in this study not all types of lymphoid PID could be studied in large series, implying that more cases of the rare diagnostic PID categories should be evaluated. This will be a continuous process to further support the clinical use of the proposed PID tubes; the EuroFlow Consortium will continue to contribute to this process.

Based on our experience in the leukemia and lymphoma field, we believe that the provided standardized strategies, tools, and reference databases are cost-effective and can easily be implemented, not only in specialized PID reference centers, but in any medical immunology laboratory equipped with an 8-color flow cytometer in any PID center in the world. However, it should be noted that standardized flow cytometry, although critical for PID evaluation, does not replace and has to be coupled to immunological functional and genetic testing in order to reach a final diagnosis in most cases.

## Ethics Statement

The study was approved by the local ethics committees of the participating centers [University of Salamanca, Salamanca, Spain (USAL CSIC 20-02-2013); Charles University, Prague, Czech Republic (15-28541A); Erasmus MC, Rotterdam, The Netherlands (MEC-2013-026); University Hospital Ghent, Belgium (B670201523515); and St. Anne's University, Brno, Czech Republic (METC 1G2015)].

## Author's Note

All authors wish to stress that they are scientifically independent and have full freedom to act without any obligation to industry other than scientific advice to companies in the context of licensed patents. The selection of antibodies by the EuroFlow consortium is always explicitly based on quality, relevance, and continuous availability. Consequently all proposed antibody panels consist of mixtures of antibodies from many different companies (see Tables 1–4).

## Author Contributions

JvD, MvdB, TK, MP-A, MvZ, and AO contributed conception and design of the study. TK, MP-A, EM, MV, EL-G, MW, A-KK, JP, AS, and EB performed the data acquisition and data analysis. MP-A and EB organized the database. JvD, MP-A, and AO wrote the manuscript. All authors contributed to manuscript revision, read, and approved the submitted version.

### Conflict of Interest Statement

JvD, MvdB, TK, MP-A, MV, EL-G, A-KK, MvZ, EB, and AO each report being one of the inventors on the EuroFlow-owned patent PCT/NL 2015/050762 (Diagnosis of primary immunodeficiencies). The Infinicyt software is based on intellectual property (IP) of some EuroFlow laboratories (University of Salamanca in Spain and Federal University of Rio de Janeiro in Brazil) and the scientific input of other EuroFlow members. All above mentioned intellectual property and related patents are licensed to Cytognos (Salamanca, ES), which company pays royalties to the EuroFlow Consortium. These royalties are exclusively used for continuation of the EuroFlow collaboration and sustainability of the EuroFlow consortium. JvD and AO report an Educational Services Agreement from BD Biosciences (San José, CA) and a Scientific Advisor Agreement with Cytognos; all related fees and honoraria are for the involved university departments at Leiden University Medical Center and University of Salamanca. The remaining authors declare that the research was conducted in the absence of any commercial or financial relationships that could be construed as a potential conflict of interest.
